# Altered Olfactory Adaptation in the Primary Olfactory Cortex in Subjective Cognitive Decline: A Task‐based Functional Magnetic Resonance Imaging Study

**DOI:** 10.1002/alz70856_098565

**Published:** 2025-12-24

**Authors:** Xi Wu, Bing Zhang

**Affiliations:** ^1^ Nanjing Drum Tower Hospital, Affiliated Hospital of Medical School, Nanjing University, Nanjing, China, Nanjing, Jiangsu, China; ^2^ Nanjing Drum Tower Hospital, Affiliated Hospital Clinical College of Nanjing Medical University, Nanjing, Jiangsu, China

## Abstract

**Background:**

Previous studies have shown that Alzheimer's disease (AD) is linked to specific activation patterns in the primary olfactory cortex (POC) as observed through functional MRI (fMRI). As the stimulus concentration increases, both mild cognitive impairment (MCI) and AD patients exhibit sustained activation, suggesting impaired olfactory habituation in these groups. However, current research on olfactory adaptation has not yet explored its effects in the subjective cognitive decline (SCD) population.

**Method:**

A total of 181 right‐handed participants were enrolled, including 60 NCs, 69 individuals with SCD, and 52 with MCI. The activation changes of POC were analyzed across four odor concentrations (0.032%, 0.1%, 0.32%, 1.0%) and phases (114s, 222s, 330s, 438s) to identify distinct olfactory adaptation patterns. Plasma biomarker levels were compared between the normal and abnormal olfactory adaptation groups.

**Result:**

The number of POC activated β×voxels in the SCD and MCI groups was significantly lower compared to the NC group. When re‐grouping the participants into three groups (NC, SCD, MCI) based on the normality of the POC olfactory adaptation pattern, chi‐square tests revealed that NC participants were predominantly in the normal olfactory adaptation group, while SCD and MCI participants were primarily in the abnormal POC olfactory adaptation group. Mediation analysis indicated that the number of POC activated voxels fully mediated the relationship between olfactory identification ability and memory cognition domains. Additionally, mean plasma levels of *p*‐tau217 and NfL were significantly higher in the abnormal olfactory adaptation group compared to the normal group.

**Conclusion:**

The study demonstrated the role of olfactory adaptation patterns in the early stages of AD, especially in SCD.

